# Experience With Reverse Sural Flap to Cover Defects of the Lower Leg and Foot

**DOI:** 10.5812/kowsar.22517464.3276

**Published:** 2012-01-15

**Authors:** Ali Ebrahimi, Nasrin Nejadsarvari, Ehsan Shams Koushki

**Affiliations:** 1 Trauma Research Center, Baqiyatallah University of Medical Sciences, Tehran, IR Iran; 2 Tehran University of Medical Sciences, Imam Khomeini Hospital, Tehran, IR Iran; 3 Trauma Research Center, Baqiyatallah University of Medical Sciences, Tehran, IR Iran

**Keywords:** Sural Nerve, Surgical Flaps, Wounds, Gunshot, Lower Extremity

## Abstract

**Background:**

Coverage of traumatic soft-tissue defects in the lower limb is a common procedure.

**Objectives:**

The purpose of this prospective case series study was explore the capacity of the perforator-based sural flap in reconstruction surgery of patients with high velocity gunshot wounds in the distal third of the leg and heel pad of the foot.

**Patients and Methods:**

A prospective case series study was undertaken to assess the sural fasciocutaneous flap carried out in our hospital, from 2010 to 2011. This case series study comprised eight patients, seven men and one woman with an average age of 35 years (19-55) and with a mean follow-up duration of 13 months (6-24 months). All patients had a history of a gunshot wound in distal part of the leg and heel pad of the foot with large soft-tissue defects; treatment was done using the reverse sural flap.

**Results:**

We performed reverse sural flaps in eight gunshot patients, to cover the defects of the lower leg and foot. Surgical site infection observed in one patient (12.5%) was treated successfully with antibiotic therapy. The reverse sural flap provided a satisfactory coverage for gunshot defects in all the patients.

**Conclusions:**

Reverse sural flap is a useful and versatile reconstructive method in patients with gunshot wounds of the lower leg and foot.

## 1. Background

Coverage of soft-tissue defects in the lower limb is a common procedure due to the increase incidence of trauma. In 1983, Donski and Fogdestam described a fasciocutaneous flap divided from the sural region. This was a basis for the reverse sural artery flaps (RSA). the main purpose of this article is to present our prospective experience with treatment of patients who have undergone the RSA flap for coverage of lower leg and heel defects due to gunshot injuries. The vascular pedicle of this reverse flap is on the septocutaneous perforating vessels that arise from the peroneal artery. These perforating arteries are most likely to be located in a region four to seven centimeters proximal to the lateral malleolus the ideal pivot point for a pedicle flap1.(1) One of the longest perforators is usually located in the four to five centimeter region above the lateral malleolus which allows this location to be considered a good site for a pivot point (2-4).

The venous drainage of this flap is important as well. Imanishi et al (5), discovered a small caliber network of veins that surround the sural nerve, which allow the bypass of valves of the lesser saphenous vein. For a long time, free flap transfer was the operation of choice in cases where the local tissues were severely damaged.

## 2. Objectives

The purpose of this prospective case series study is to explore the capacity of the perforator-based sural flap in reconstructive surgery of patients with high velocity gunshot wounds in the distal third of the leg and heel pad of the foot.

## 3. Patients and Methods

**Figure 1.**
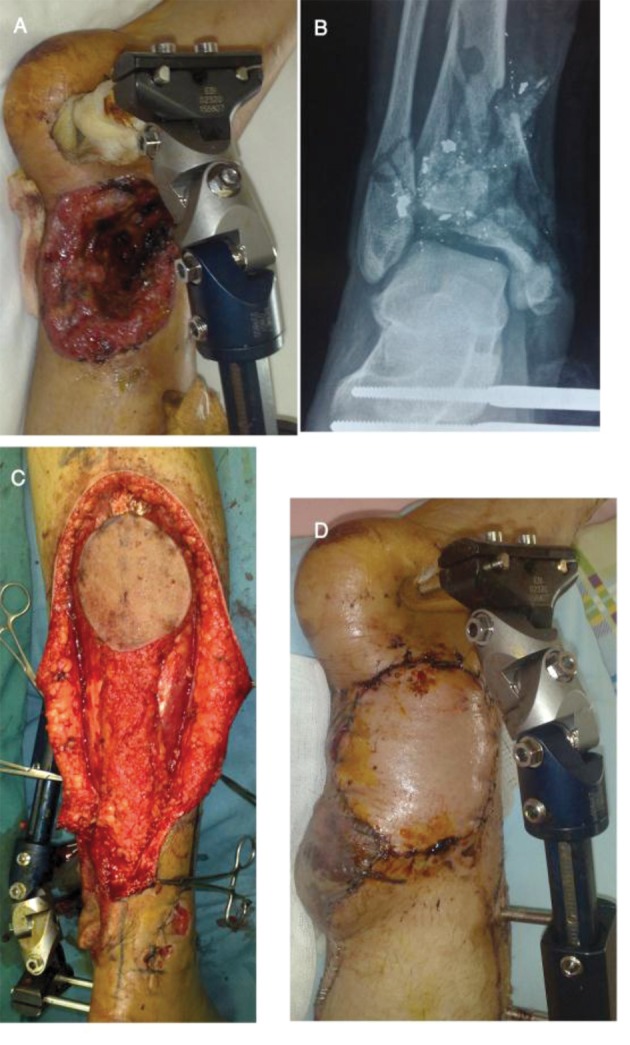
A thirty-five-year-old patient with defects of the lower leg and tibial bone: A-Before reconstruction, B- X-ray view, C-Intra-operative view, D- Follow-up at one month.

**Figure 2.**
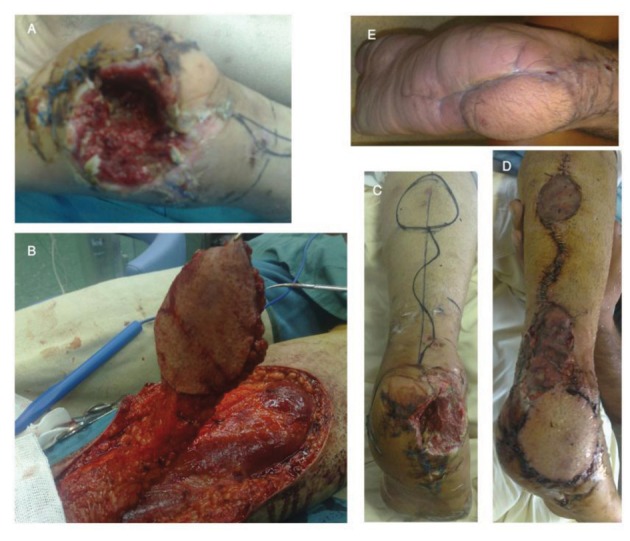
A twenty year-old patient with traumatic calcanectomy after a mine explosion. A-before reconstruction, B&C-intra-operative view, DEarly post-operation, E-Follow-up at six months.

We present a prospective case series study of the sural fasciocutaneous flap carried out in our hospital between 2010 to 2011. This case series study comprised eight patients, seven men and one woman with an average age of 35 years (19-55) and with a mean follow-up duration of 13 months (6-24 months). All patients had a history of a gunshot wound in distal of the leg or heel pad of the foot and large soft-tissue defects; in two patients we had defects of the tibial bone due to crushing effect of the bullet (Figure 1) and in another patient we had post-traumatic calcanectomy after mine explosion (Figure 2). The remaining five patients, had bone exposed soft-tissue defects in the distal of the leg. This retrograde fasciocutaneous flap was based on blood supply provided from fasciocutaneous perforators of the peroneal artery; fasciocutaneous perforators from the posterior tibial artery, venocutaneous perforators from the lesser saphenous vein, and neuro-cutaneous perforators from the sural nerve (6, 7). The classical arterial supply of the distally based sural flap is provided by septo-cutaneous perforators arising from the peroneal artery. On average, each leg has three to six perforators located 4 to 7 centimeters proximal to the lateral malleolus.

### 3.1. Surgical Technique

We design this fasciocutaneous flap in the proximal posterior region of the leg and the pivot point for this flap should be 5cm posterior and superior to the lateral malleolus, (Figure 1 and 2) (4, 8). Distance between island portion and defect is dependent on the site of the defect and the pivot point. We must be careful about any pressure or tension on the pedicle of the flap. Doppler Ultra-Sonography may be used to identify perforator vessels to help in planning the pivot point. Dissection of the flap must be subfascial and sural nerve and lesser saphenous vein should be ligated and elevated with the flap. The skin overlying the fascial pedicle is then undermined to the pivot point. We chose the width of the pedicle about 2-3 centimeters according to the sural nerve pathway (Figure 1 and 2). We did not use tourniquet during flap elevation and insetting; before flap coverage, debridement of the wound was done and all necrotic tissues removed. To repair the donor site, we used split-thickness skin graft if the defect width was more than 6 centimeters. Also we used small split thickness skin graft for coverage of the pedicle pivot point. We explained probable complications to all patients and Informed consents were obtained from all the patients before intervention.

## 4. Results

We performed reverse sural flaps in eight patients, for coverage the defects of gunshot wound in the lower leg and in the foot. After operation, patients had leg elevation to reduce venous congestion and splint immobilization recommended for two weeks. The success rate was 100%. Surgical site infection observed in one patient (12.5%) that was treated successfully with antibiotic therapy (flap remained viable). In one case that had tibial bone defect, reconstruction with this flap was done and thereafter we referred this patient to an orthopedic surgeon for bone graft (eight weeks post reconstruction). The flap provided a satisfactory coverage for such defects in all the patients. We had no failure or necrosis in these patients. Slight venous congestion of the flap occurred in some patients but it resolved with leg elevation. The average flap size was 7-10 centimeters. Wound debridement was done before operation. In one patient, we applied external fixation for bone stability, and in all patients fascia was included in the flap. This flap is a suitable choice for distal of the leg and hind-foot defects due to high velocity gunshot wounds. Arterial perforators above lateral malleolus are intact although they are near the injury site; the blast effect of high velocity gunshots has no deteriorating effects on these small fasciocutaneous perforators.

## 5. Discussion

Reverse sural flap is a useful and versatile reconstructive method in patients with soft tissue defects of the lower leg and foot. In practice, the flap size and pivot point position are determined by the geometric contour of the defects. The flap should be free of pressure and other mechanical forces.(9) This flap has the largest arc of rotation compared to the regional flaps and does not require sacrifice of any major artery, and moderate to large sized defects can be covered adequately.(10) We must place a light dressing on the flap and limb elevation is crucial in the prevention of venous congestion. There is a large variety of muscular or pedicled flaps for reconstruction of lower limb soft tissue defects, lateral supramalleolar skin flap, posterior tibial perforator flap, sural flap and free flaps are the main reconstructive options in lower limb and foot defects. Free flaps are advised for extensive skin defects or in cases where poor distal vascularity of the leg precludes the reliability of a distally based fasciocutaneous flaps (11-13). Lateral supramalleolar flap offers a range of coverage similar to that of the sural flap but the dissection is more difficult than for a sural flap. Some authors stated that the distally based sural flap is more reliable than the lateral supramalleolar flap, especially regarding venous congestion. In addition; sural flap is useful for coverage of weight bearing areas, a lateral supramalleolar skin flap is not recommended for coverage in this area (14).

In all cases, patency of common peroneal artery was established with Doppler probe before operation. High velocity gunshot wounds have special characteristics. Necrotic tissues and crushing of tissue adjacent to central ulcer need serial debridement for preparation of ulcer reconstruction. This flap is dependent on reliable blood flow through the peroneal artery and it is necessary in war injured patients to determine the patency of this artery before elevation of this flap. Disadvantages of this flap are limitation of pedicle rotation, cosmetic appearance of the scar at the donor site, dependency on patient peroneal artery, and sacrifice of sural nerve during reconstruction, but this limitation seems of little importance in the context of limb salvage (15). Significant contraindication for a reverse sural flap consists of active soft tissue infection and absence of in-line flow to the peroneal artery (16). Our success rate in this study was 100% if we do not consider temporary venous congestion as a complication. Also in one patient, we had surgical recipient site infection (12.5%) that was treated by antibiotic therapy combined with frequent dressing change. We recommend this flap for reconstruction of problematic wounds due to gunshot injuries in distal third of the leg and foot.

Another option in this area is the posterior-tibial perforator flap. This flap has been used as a reliable method in lower limb defects, especially in reconstruction of chronic Achilles tendon defects.(17) The advantages of the sural flap compared to other covering methods, are the simplicity of the design and dissection of the pedicle flap that can be carried out with loupe magnification and without the need for microsurgical instrumentation. This is a well vascularized islet flap offering the possibility of covering a broad range of areas with cutaneous defects in the distal of tibia, heel and up to the rear foot (18). The reverse sural flap is reliable and easy to raise, should not depend on microsurgery and should not sacrifice a major artery or nerve (19).

This flap has been used for open fractures, osteomyelitis, pressure ulcers, diabetic ulcers and trauma. Donor sites have been closed primarily when defect size was between 4-6 centimeters.(9) In this case series study, we used this fasciocutaneous flap to cover gunshot wounds in the lower leg and heel pad which were not reconstructed by simple methods. In our eight patients, the overall success rate was 100%, without any significant complication. Our sample size was small when compared with other studies, but this study reports our clinical experience, using reverse sural in reconstruction of the gunshot wounds of the lower extremity. This flap is a suitable choice for difficult gunshot wounds in distal of the leg and hind-foot when peroneal artery is patent. Reverse sural flap can be an alternative method for free tissue transfer in reconstruction of the lower extremity defects.

## References

[1] Yang D, Morris SF (2002). Reversed sural island flap supplied by the lower septocutaneous perforator of the peroneal artery.. Ann Plast Surg..

[2] Masquelet AC, Romana MC, Wolf G (1992). Skin island flaps supplied by the vascular axis of the sensitive superficial nerves: anatomic study and clinical experience in the leg.. Plast Reconstr Surg..

[3] Chen SL, Chen TM, Chou TD, Chen SG, Wang HJ (2002). The distally based lesser saphenous venofasciocutaneous flap for ankle and heel reconstruction.. Plast Reconstr Surg..

[4] Hasegawa M, Torii S, Katoh H, Esaki S (1994). The distally based superficial sural artery flap.. Plast Reconstr Surg..

[5] Imanishi N, Nakajima H, Fukuzumi S, Aiso S (1999). Venous drainage of the distally based lesser saphenous-sural veno-neuroadipofascial pedicled fasciocutaneous flap: a radiographic perfusion study.. Plast Reconstr Surg..

[6] Batchelor JS, McGuinness A (1996). A reappraisal of axial and nonaxial lower leg fascial flaps: an anatomic study in human cadavers.. Plast Reconstr Surg..

[7] Nakajima H, Imanishi N, Fukuzumi S, Minabe T, Aiso S, Fujino T (1998). Accompanying arteries of the cutaneous veins and cutaneous nerves in the extremities: anatomical study and a concept of the venoadipofascial and/or neuroadipofascial pedicled fasciocutaneous flap.. Plast Reconstr Surg..

[8] Hollier L, Sharma S, Babigumira E, Klebuc M (2002). Versatility of the sural fasciocutaneous flap in the coverage of lower extremity wounds.. Plast Reconstr Surg..

[9] Chen SL, Chen TM, Chou TD, Chang SC, Wang HJ (2005). Distally based sural fasciomusculocutaneous flap for chronic calcaneal osteomyelitis in diabetic patients.. Ann Plast Surg..

[10] Cheema TA, Saleh ES, De Carvalho AF The Distally Based Sural Artery Flap for Ankle and Foot Coverage.. The Journal of Foot and Ankle Surgery..

[11] Benito-Ruiz J, Yoon T, Guisantes-Pintos E, Monner J, Serra-Renom JM (2004). Reconstruction of soft-tissue defects of the heel with local fasciocutaneous flaps.. Ann Plast Surg..

[12] Costa-Ferreira A, Reis J, Amarante J (2005). Reconstruction of soft-tissue defects of the heel with local fasciocutaneous flaps.. Ann Plast Surg..

[13] Erdmann D, Gottlieb N, Humphrey JS, Le TC, Bruno W, Levin LS (2005). Sural flap delay procedure: a preliminary report.. Ann Plast Surg..

[14] Price MF, Capizzi PJ, Watterson PA, Lettieri S (2002). Reverse sural artery flap: caveats for success.. Ann Plast Surg..

[15] Yilmaz M, Karatas O, Barutcu A (1998). The distally based superficial sural artery island flap: clinical experiences and modifications.. Plast Reconstr Surg..

[16] Baumeister SP, Spierer R, Erdmann D, Sweis R, Levin LS, Germann GK (2003). A realistic complication analysis of 70 sural artery flaps in a multimorbid patient group.. Plast Reconstr Surg..

[17] Cavadas PC, Landin L (2006). Reconstruction of chronic Achilles tendon defects with posterior tibial perforator flap and soleus tendon graft: clinical series.. Plast Reconstr Surg..

[18] Rios-Luna A, Villanueva-Martinez M, Fahandezh-Saddi H, Villanueva-Lopez F, del Cerro-Gutierrez M (2007). Versatility of the sural fasciocutaneous flap in coverage defects of the lower limb.. Injury..

[19] Hong G, Steffens K, Wang FB (1989). Reconstruction of the lower leg and foot with the reverse pedicled posterior tibial fasciocutaneous flap.. Br J Plast Surg..

